# Apparent metabolizable energy, growth performance and carcass traits of Japanese quail fed select modern grain sorghum varieties

**DOI:** 10.5713/ab.22.0047

**Published:** 2022-06-28

**Authors:** A. H. Moritz, S. K. Krombeen, J. Presgraves, M. E. Blair, R. E. Buresh, W. C. Bridges, M. Arguelles-Ramos, T. A. Wilmoth

**Affiliations:** 1 Department of Animal and Veterinary Sciences, Clemson University, Clemson, SC 29634, USA; 2 United Animal Health, Sheridan, IN 46069, USA; 3 Novus International, Inc., St. Charles, MO 63304, USA; 4 Department of Mathematical and Statistical Sciences, Clemson University, Clemson SC 29634, USA

**Keywords:** Apparent Metabolizable Energy, Carcass Traits, Grain Sorghum, Japanese Quail, Performance, Tannin-free

## Abstract

**Objective:**

This study was performed to determine the apparent metabolizable energy (AME_n_) content of tannin-free red/bronze, white/tan and U.S. No. 2 varieties of grain sorghum for feeding Japanese quail and validate their nutrient profile by evaluating effects on performance and carcass traits with full-substitution of corn.

**Methods:**

Experiment 1 determined the AME_n_ content of red/bronze, white/tan, and U.S. No. 2 grain sorghum varieties fed to mixed-sex Japanese quail (*Coturnix japonica*) (n = 314) at 3 and 6-weeks of age. Analyses were based on a 2×4 factorial treatment design with age and grain types defining the treatments, and a randomized complete block experiment design with cage and trials defining the block. AME_n_ values were validated by evaluating the performance and carcass traits of Japanese quail (n = 644) from 1 to 40 days of age in Experiment 2 with birds were fed 1 of 4 complete diets. Statistical analyses were conducted on performance data and select individual carcass trait measurements.

**Results:**

Determined AME_n_ values at 3-weeks of age were 3,524±122.03 (red/bronze), 3,252± 122.03 (white/tan), and 3,039±123.44 (U.S. No. 2) kcal/kg. At 6-weeks of age, determined AME_n_ were 3,373±297.35 (red/bronze), 3,279±297.35 (white/tan), and 2,966±298.64 (U.S. No. 2) kcal/kg. Carcass traits showed live body weight (p = 0.0409) and hot carcass weight (p = 0.0234) were greatest in U.S. No. 2; however, carcass yield (p<0.0001) was lowest. No significant differences were observed among treatments for feed intake, feed conversion ratio, breast weight and breast yield (p>0.05).

**Conclusion:**

These studies demonstrated that tannin-free grain sorghum varieties may be a potential alternative to corn in quail diets while maintaining growth performance and carcass parameters.

## INTRODUCTION

Increasing feed costs and demand to meet the global food supply have driven the poultry industry to reduce the heavy dependence on corn and seek alternative feedstuffs. Grain sorghum is an alternative grain to corn due to its similar metabolizable energy and its adaptations to drought and varying soil types [1]. Considering its environmental adaptability, it may be a viable alternative to grow in locations where the soil is drier, drought conditions exist, and coincides with a heavy poultry production area, specifically, the southeastern United States (U.S.). Although widely known that the gamebird industry is significantly smaller in production than the broiler industry, consumer trends show increased demand in alternative poultry meat, including quail [2]. Such peaked demand has led to this specialty market with increasing popularity from consumers seeking healthier meat alternatives lower in fat and higher in protein [3]. The largest Japanese quail producer in the U.S., producing more than 6 million birds annually, is located in the southeastern U.S. However, currently, grain sorghum and poultry markets are geographically separate from each other, with a majority of grain production concentrated west of the Mississippi River and broiler production located heavily in the Southeastern region of the U.S. [4]. As a result of this geographic disconnect between the two markets, grain sorghum is used less frequently in poultry diets than corn.

Additionally, inconsistencies commonly associated with tannins in grain sorghum have invoked a negative perception for using it as an alternative; however, evaluation of low tannin grain sorghum varieties have shown to be an adequate substitution without affecting growth in broilers [5,6]. Genetic modifications, accomplished through selective breeding have led to tannin-free grain sorghum for animal feed use in the U.S. today. The most prevalent varieties of modern, tannin-free grain sorghum varieties and those used in the following study include red/bronze, white/tan, and U.S. No. 2 (Personal communication, 2018). However, limited data are available to support the use of tannin-free varieties as an alternative feedstuff to corn at full-substitution levels and are especially limited for use in gamebird feeds, mainly Japanese quail.

Therefore, validating the nutritional impact of tannin-free grain sorghum varieties on the growth, health, and product meat quality of gamebirds is necessary to support the use of sorghum as an alternative to corn in poultry feeds. Indeed, determining the apparent metabolizable energy (AME_n_) value of each of these modern grain sorghum varieties is a critical factor in diet formulation for full replacement of corn in quail diets. Evaluation of the nutritional composition of tannin-free grain sorghum will also elucidate perceived issues and offer commercial poultry nutritionists up-to-date specifications for practical use in feed formulation for tannin-free grain sorghum. Therefore, the objective of this study was to determine the AME_n_ content of red/bronze, white/tan, and U.S. No. 2 varieties of grain sorghum for feeding commercial Japanese quail and validate its nutrient profile by evaluating the effect of full substitution of corn with grain sorghum on growth performance and carcass traits.

## MATERIALS AND METHODS

All procedures were approved by the Clemson University Institutional Animal Care and Use Committee (AUP #2017-051).

### Birds and husbandry

#### Experiment 1. Apparent metabolizable energy

Two trials with identical procedures were conducted for a 1 to 40 day of age grow-out period. Trials evaluated the AME_n_ response of mixed-sex commercial Japanese quail (*Coturnix japonica*) (n = 314) at 3-weeks of age (19 to 21 days) and 6-weeks of age (36 to 38 days). In each trial, quail (n = 157) were housed in a solid-sided research house and randomly distributed (5 birds per cage) in heated battery brooder cages, 34 cm×98 cm (Petersime, Zulte, Belgium). Each starter battery cage was provided with a trough feeder and quart jar waterer. In both trials, the temperature of the house was 37°C at placement and gradually decreased to reach 27°C with 16 hours of light to 8 hours of dark (16 L:8 D) throughout the study.

#### Experiment 2. Growth performance

One trial evaluated the growth performance and carcass traits of mixed-sex Japanese quail (*Coturnix japonica*) (n = 644) 1 to 40 days of age. Quail were housed in a curtain-sided broiler house, group weighed (n = 14 birds) per cage on day 1 of age, randomly distributed into 46 rabbit wire hutches, 76 cm×91 cm, (Pet Lodge; Miller Manufacturing, Glencoe, MN, USA), and placed inside floor pens, 183 cm×305 cm, containing wood shavings as bedding material. Each cage was provided with a trough feeder and quart jar waterer. The temperature of the house was 37°C at placement and gradually decreased to reach 23°C with a daily lighting program of 16 hours of light to 8 hours of dark (16 L:8 D) throughout the study.

### Tannin analysis

Varieties of grain sorghum used in this experiment were analyzed for tannin content to ensure zero tannin content before use in experimental diets. An acid-butanol assay (University of Miami, Oxford, OH, USA) for proanthocyanidins was conducted on red/bronze, white/tan, and the U.S. No. 2 grain sorghum varieties. In this assay, acid was added to a sample of each grain to yield a colored product (known as cyanidin) or a colorless product (known as catechin) if tannins were present or absent, respectively [7]. The colored product has an absorbance peak at 550 nm and is characteristic of a high tannin grain [7]. The absorbance measured for each grain sorghum variety for this experiment yielded no product at 550 nm compared to the pure sorghum with spectra at 550 nm; thus, no tannins were present in the varieties used for this experiment. Additional confirmation for the presence of tannins in the red/bronze variety, which is more commonly known to contain higher tannin content, was tested at a high and low concentration of tannin to ensure that there were no traces of tannins in larger sample sizes. Results showed similar spectra for both concentrations with no peak at 550 nm, indicating no tannin traces in any of the samples.

### Treatments

Three modern varieties of grain sorghum commonly grown in the southeastern U.S., red/bronze, white/tan, and U.S. No. 2, were obtained from Florida and North Carolina and used for all diets in Experiments 1 and 2. The red/bronze and white/tan grain sorghum varieties were identity preserved (contained a single variety), while U.S. No. 2 was a red/bronze based variety that may have contained other mixed grain sorghum varieties. Nutrient and gross energy (GE) analyses of grain sorghum used in the experimental diets are shown in Table 1. All whole grain sorghum was ground through a hammer mill (Premier 1 Supplies; Washington, IA, USA) with a 4-mm sieve.

#### Experiment 1

Japanese quail were fed starter/grower mash feed ad-libitum and formulated based on an industry standard supplied by a commercial nutritionist. All birds were fed a corn-based acclimation diet on days 1 to 3. On day 4, birds were randomly assigned 1 of 4 corn basal diets with 20% of the calories for the GE of corn replaced by the equivalent calories of the respective GE of grain sorghum (red/bronze, white/tan, or U.S. No. 2; Table 1) or dextrose for the control. Dextrose was used as a reference ingredient due to its known energy content that would replace 20% of the calories for the GE of corn removed from a corn-based diet to replace each respective test ingredient (i.e., grain sorghum). The classical basal substitution method [8] was modified in this study to formulate treatment diets based on the GE content of corn to be replaced with an energetically equivalent amount of grain sorghum in order to target a more practical approach and evaluation for future use by commercial nutritionists. The ingredient composition, nutrient analyses, and AME_n_ for the basal and complete test diets are shown in Tables 2 and 3. Each trial included 10 replicates of dextrose-control diet (5 birds per cage), and each of the three grain sorghum treatment diets included 18 replicates (5 birds per cage) over the grow-out of 1 to 40 days of age.

#### Experiment 2

AME_n_ values (Table 4) determined for grain sorghum varieties in Experiment 1 were used in diet formulation for the growth performance trial. Japanese quail were fed one phase constituting a starter/grower diet mash ad-libitum from 1 to 40 days of age. Table 5 shows the ingredient compositions and nutrient analyses for Japanese quail diets. All birds were fed a corn-based acclimation diet on days 1 to 3. On day 4, birds were randomly assigned to 1 of 4 treatments including a full inclusion corn diet serving as the control (6 replicates, 14 birds per pen) and full inclusion of red/bronze, white/tan, and U.S. No. 2 grain sorghum (10 replicates, 14 birds per pen).

### Excreta collection and measurements

#### Experiment 1

Excreta collection and other measurements for AME_n_ determination of grain sorghum was determined in Japanese quail at 3-weeks of age (19 to 21 days) and 6-weeks of age (36 to 38 days). At the end of each collection period, feed disappearance and total excreta weight were measured. A 30-g sample of feed and excreta was analyzed for dry matter, GE, and nitrogen content with a bomb calorimeter and combustion N analyzer at the University of Georgia Feed, Environmental and Water (FEW) Laboratory (Athens, GA, USA). Feed intake (FI), excreta weight, GE, and nitrogen (N) content results were used to calculate the nitrogen corrected AME_n_ of grain sorghum using the difference method by MacLeod et al [9]:


(1) $$\text{Treatment }{\text{AME}}_{\text{n}}{\text{AME}}_{\text{n}}=[\text{GEI}-\text{GEE}]-[8.73\times (\text{NI}-\text{NE})]÷\text{FI}$$

(2) $$\text{Sorghum }{\text{AME}}_{\text{n}}{\text{AME}}_{\text{n grain sorghum}}={\text{AME}}_{\text{n basal}}+({\text{AME}}_{\text{n treatment}}-{\text{AME}}_{\text{n basal}})÷(\text{proportion of added grain sorghum})$$

where GEI, gross energy intake; GEE, gross energy output in excreta; NI, nitrogen intake from the diet; NE, nitrogen output from excreta; FI, feed intake; and 8.73 = nitrogen correction factor from previous research [10].

### Performance and carcass measurements

#### Experiment 2

Birds were group weighed (kg/cage), and feed disappearance was measured weekly on days 1, 8, 15, 22, 29, 36, and 40. Weekly measures of mean body weight (BW) and feed disappearance were used to calculate mean FI and feed conversion ratio (FCR) per treatment:


(3) $${\text{FCR}}_{not\;adjusted\;for\;mortality}=\frac{\text{mean FI }(1-40\;\text{days of age})}{\text{mean BWG }(1-40\;\text{days of age})}$$

At 40 days of age, feed was withdrawn, and birds fasted for 12-hours before slaughter, an accepted industry standard procedure. All birds were group weighed per pen and treatment, and 6 birds from each replicate were randomly selected, group weighed for total live body weight (LBW), and transported to the meat laboratory at Clemson University for slaughter and further processing. Birds were euthanized via cervical dislocation, and eviscerated whole carcasses were individually weighed to measure hot carcass weight (HCW) and carcass yield (CY) (Table 7). All carcasses were placed on ice and stored at 3°C for 24 hours. Then, whole carcasses were drained to remove excess water and weighed to obtain chilled carcass weight. Next, the front half of each carcass with pectoral major and minor muscles (bone-in) were removed and individually weighed to measure breast weight (BrW) and breast yield (BrY). Carcass and breast yields were calculated as a percentage of HCW.

### Statistical analyses

#### Experiment 1

The initial analyses were based on a 2×4 factorial treatment design with age (3-weeks and 6-weeks) and grain types (dextrose-control, red/bronze, white/tan, and U.S. No.2 grain sorghum) defining the treatments, and a randomized complete block experiment design with cage and trials defining the block. The model effects were assessed with analysis of variance (ANOVA) based on the treatment and experiment designs. Fisher’s protected least significant difference procedure was used to determine specific differences among the treatment means. Statistical significance was defined when p values were less than 0.05. All statistical calculations were performed using JMP Pro version 15.2.0 (SAS Institute Inc., Cary, NC, USA).

#### Experiment 2

The data analysis was based on a one factor treatment design with the grain types (dextrose-control, red/bronze, white/tan, and U.S. No. 2) defining the fixed treatments, and a completely randomized experiment design with pen designated as the random experimental unit of 14 Japanese quail per pen (n = 644 birds). Statistical analyses were conducted on performance data of mean pen-grouped birds, including mean BW, FI, FCR, and select individual bird carcass trait measurements (HCW, CY, BrW, and BrY) using an ANOVA based on the treatment and experimental designs. Fisher’s protected least significant difference procedure was performed to determine specific differences among treatment means. Statistical significance was defined when p values were less than 0.05. All statistical calculations were performed using JMP Pro version 15.2.0 (SAS Institute Inc., Cary, NC, USA).

## RESULTS AND DISCUSSION

### AME_n_ determination

In Experiment 1, the determined AME_n_ for grain sorghum at 3-weeks of age presented in Table 4 were 3,524±122.03 (red/bronze), 3,252±122.03 (white/tan), and 3,039±123.44 (U.S. No. 2 grain sorghum) kcal/kg. Red/bronze had the highest AME_n_ compared to dextrose-control, white/tan, and U.S. No. 2 (p<0.0001). At 6-weeks of age, the determined AME_n_, shown in Table 4, were 3,373±297.35 (red/bronze), 3,279±297.35 (white/tan), and 2,966±298.64 (U.S. No. 2) kcal/kg. The highest determined AME_n_ was the red/bronze grain sorghum, while white tan was intermediate when compared to U.S. No. 2 (p = 0.0322), but not significantly different from red/bronze. No significant differences were observed in the 72-h FI at 3 and 6-weeks of age (p>0.05). No differences were observed in mortality (p>0.05) with an overall mean of 5.5%.

AME_n_ values determined in Experiment 1 for meat-type quail were not affected by age from 3 to 6-weeks of age (p = 0.5147) or when age and treatment interaction were anlayzed (p = 0.8746). This indicates no effect of age on AME_n_ value and that a single-phase feeding program is an adequate recommendation for quail as was provided as a starter/grower diet. In a previous study by Moritz et al [11] determining the AME_n_ of grain sorghum for broilers, values varied by feed phase; mean AME_n_ values of modern grain sorghum varieties for broilers in the grower diet-phase were determined as 3,336 (red/bronze), 4,000 (white/tan), and 3,341 (U.S. No. 2 grain sorghum) kcal/kg (as-fed), respectively. In the finisher diet-phase, average AME_n_ values were determined as 3,001 (red/bronze), 3,599 (white/tan), and 3,705 (U.S. No. 2 grain sorghum) kcal/kg (as-fed), respectively. Other studies in comparison, show AME_n_ values of 2,790 and 2,651 kcal/kg (DM) for tannin-free, white and red sorghum varieties, respectively, in broiler chickens from 7 to 28 days of age [12]. Additional variations in feed intake, excreta measurements, and AME_n_ methodology can also influence AME_n_ values [13].

Species, age, and sex of birds are a few variables known to cause variability in energy metabolism [14]. Feed intake in Experiment 1 was not affected by AME_n_, suggesting no influence of treatment when 20% of the GE of corn is substituted by grain sorghum even though there were varying AME_n_ values across treatments at 3 and 6-weeks of age. Muniz et al [15] and Wilson et al [16] reported a negative correlation between FI and AME_n_ of which, birds consume more feed of lower AME_n_ and less feed of higher AME_n_. However, this finding was not in agreement with the lack of difference in feed intake of Experiment 1. This may also suggest that quail do not respond as similarly to energy levels as would broilers to adjust their feed consumption to meet their energy requirement [16]. The lower AME_n_ observed in Experiment 1 in the U.S. No. 2 grain sorghum may be a result of a lower GE value compared to the other GE analyses for grain sorghum varieties (Table 1); therefore, the energy available and used by the bird was numerically lowest for the U.S. No. 2 treatment. Previous studies by Moritz et al [11] and Khalil et al [17] observed lower AME_n_ values for tannin-free and low-tannin grain sorghum, respectively with increasing age attributed by increased FI and as a result, increased rate of passage and digestion. In addition, starch digestibility has been reported to have a negative correlation with increasing FI and reduce AME_n_ [Svihus]. Similiarly, in this present study, nutrient analyses (Table 1) show U.S. No. 2 grain sorghum with the highest crude fiber content which may be a result of lower AME_n_ values compared to other treatments. Furthermore, additional variations in the nutrient composition of an ingredient can be a result of its region, environment, and season in which it is grown, and such variability can influence the energy density of a feed; thus, diet formulation [18,19].

### Growth performance and carcass traits

In Experiment 2, growth performance in Table 6 showed the greatest BW observed in the U.S. No. 2 grain sorghum treatment compared to corn, red/bronze, and white/tan treatments (p = 0.0031). No significant differences were observed among treatments for FI and FCR (p>0.05).

Although performance data for quail fed tannin-free grain sorghum is limited, results are in agreement with previous studies evaluating performance of quail fed tannin-free red and white grain sorghum varieties with no effect of treatment or performance [20]. Compared to broilers, Hulan and others demonstrated that low tannin grain sorghum can replace corn by 45% to 58% inclusion without detrimental effects on weight gain, feed conversion, CY, and feed intake [21]. Higher tannin grain sorghum varieties resulted in decreased bodyweight, feed intake and poorer feed conversion in broilers [17]. Dykes and Rooney [22] indicated that birds can consume both tannin and non-tannin sorghum varieties, but it has been observed that birds will eat low-tannin white sorghum before they will low-tannin red grain sorghum. They also noted that varieties containing tannins do result in adverse effects on performance and efficiency. It is widely known by researchers, nutritionists, and grain producers that previous varieties of grain sorghum are associated with tannins and have suboptimal effects on digestibility and growth performance in broilers [22]. Nevertheless, in Experiment 1 and 2, tannin-free grain sorghum varieties were acquired in the U.S. and analyzed as tannin-free (Figures 1 and 2).

The high mortality observed in Experiment 2 (Table 6), primarily in the first week of grow-out, was thought to be explained by high fumonisin mycotoxin levels detected in white/tan, red/bronze and U.S. No. 2 grain sorghum. However, based on mycotoxin analyses, the average fumonisin level detected for grain sorghum varieties was 0.463 mg/kg compared to <0.005 mg/kg of aflatoxin (B1, B2, G1, G2,), ochratoxin A, T-2 toxin, HT-2 toxin, vomitoxin, and zearalenone. No adverse effects on performance were evident in Experiment 2. According to Awad and others [23], poultry have a high tolerance specifically to *Fusarium* species like fumonisin mycotoxin which have been shown to have immunotoxic effects on gut/immune function and feed consumption when *Fusarium* toxin is >5 mg/kg. It has been shown that long-term exposure to dietary mycotoxins have previously presented adverse effects on bird performance including decreased BW and efficiency [24]. However, in a study by Swamy et al [24] growth parameters were not impacted in the finisher diet-phase when evaluating *Fusarium* mycotoxins in broilers suggesting that birds potentially adapt to mycotoxins over time. Although the high mortality observed in this current study was thought to be a result of a similar immunotoxic effect in early grow-out, the *Fusarium* toxin level was not >5 mg/kg. Therefore, the observed high, first-week mortality may have been due to poor chick quality and on-farm management practices. In a study by Yerpes et al [25], risk factors influencing high rates of first-week mortality in broiler chicks were identified and included internal factors such as chick quality dependent on hatchery management, and external factors dependent on house management and environmental factors.

While mycotoxins were analyzed and may have been one of many contributing factors to high mortality in Experiment 2, it should be noted that lower AME_n_ values for U.S. No.2 grain sorghum in Experiment 1, (Table 4) could also be due to potential mycotoxin contamination. Previous research by Nelson et al [26], found that contaminated corn with mycotoxins significantly reduced nutrient digestibility and energy utilization. Additionally, reduced grain quality can also have a significant effect on mycotoxin production [25]. As mentioned previously, grain quality and its variations in nutrient composition can exist depending on the region or environment the feedstuff is grown and sourced from which can influence energy values [19].

Carcass traits in Table 7 show LBW (p = 0.0409) and HCW (p = 0.0234) were greatest in U.S. No. 2 compared to all other treatments; however, CY (p<0.0001) was lowest in the U.S. No. 2 treatment. No significant differences were observed among treatment for BrW and BY (p>0.05).

Carcass traits are essential parameters when processing meat for retail markets [27]. Quail in the U.S. No. 2 treatment had heavier LBW and HCW, but had the lowest CY compared to other treatments. Lower CY may be due to early onset of egg laying/sexual maturity for quail in the U.S. No. 2 treatment. A previous study by Yang et al [28] compared the level of adipose tissue and its physiological changes in onset laying and pre-laying quail. Results showed that onset laying quail had heavier BWs, increased body fat and increased liver weight than pre-laying quail [28]. In this current study, the heavier weight in the U.S. No. 2 treatment may have been contributed by the higher body fat and liver weight for sexually mature quail at 39 days of age than for muscle deposition on the carcass for CY results. Not to mention, the ingredient composition and nutrient analysis for the U.S. No. 2 diet (Table 5) had a higher fat inclusion and crude fat analysis which may have influenced the observed results. Nonetheless, compared to published data, CY and BrY are consistent with what has been previously reported by Santhi and Kalaikannan in which optimal CY is between 64% to 65% and breast yield is 33% [2] at 42 days of age. Effect of sex (male or female) can influence yield [29]; however, when statistically analyzed, there was no significant effect of sex in sample birds.

## IMPLICATIONS

Current nutrient composition and performance data for grain sorghum fed to Japanese quail is inconsistent and limited with previous studies focused on high tannin varieties and their negative influence when fed to broilers. Data from this study can provide nutritionists with an updated nutrient composition of tannin-free grain sorghum and redefine negative perceptions to give producers confidence in using modern varieties for commercial quail production. AME_n_ values determined in this study should be used as a reference and not as absolute values due to nutritional variations of grain quality and the region or environment the feedstuff is grown and sourced.

## Figures and Tables

**Table 1:** Nutrient and gross energy analyses of sources of corn and modern varieties of grain sorghum (red/bronze, white/tan, and U.S. No. 2)

Proximates (%)^1)^	Dextrose	Corn	Grain sorghum variety		

			Red/Bronze	White/Tan	U.S. No. 2
Dry matter	-	84.58	87.48	89.93	84.44
Gross energy, as-fed (kcal/kg)^2)^	3,376	3,926	3,752	3,686	3,653
Ash	-	1.07	0.90	1.10	1.39
Crude fat	-	3.13	2.89	2.46	2.93
Crude fiber	-	1.50	1.80	1.70	2.20
Crude protein	-	7.52	8.87	9.33	8.65
Methionine	-	0.15	0.15	0.17	0.14
Lysine	-	0.26	0.24	0.24	0.23
Threonine	-	0.26	0.30	0.31	0.28

1) Proximate analysis was determined using the AOAC (Association of Official Analytical Chemists) method (Novus International, Inc. Laboratory Services, St. Charles, MO, USA).

2) Determined by University of Georgia Feed, Environmental and Water (FEW) Laboratory (Athens, GA, USA).

**Table 2:** Ingredient composition of basal Japanese quail diet (as-fed) from a 1 to 39 day grow-out for complete test diets

Items	Starter/grower (1 to 39 d)
Ingredient (%)	
Corn	34.61
Soybean meal, 47.5% CP	50.86
Fat, vegetable	8.15
Mono-dicalcium phosphate	2.79
Limestone	1.27
Sodium chloride	0.45
DL-methionine	0.55
L-threonine	0.18
Biolys	0.58
Choline chloride, 60%	0.27
Vitamin and trace mineral premix^1)^	0.19
Copper sulfate (5H_2_O)	0.05
Saccox 60^2)^	0.05
Calculated AME_n basal_ (DM, kcal/kg)	2,874
Calculated composition (%)	
ME (kcal/kg)	3,120
Crude Protein	27.60
Calcium	1.38
Sodium	0.20
Lysine	1.79
Methionine	0.90
Methionine+cysteine (SAA)	1.28
Total phosphorous	1.17
Available phosphorus	0.65

AME_n_, apparent metabolizable energy; ME, metabolizable energy.

1) Commercial vitamin and trace mineral premix for turkeys.

2) Salinomycin sodium, Saccox 60 (Huvepharma, Peachtree City, GA, USA).

**Table 3:** Ingredient composition and nutrient analyses of complete starter/grower Japanese quail test diets for Experiment 1 from 4 to 39 days of age (corn/dextrose, red/bronze, white/tan and U.S. No. 2) (%, as-fed basis)

Items	Starter/grower treatments			

	Dextrose-control	Red/Bronze	White/Tan	U.S. No. 2
Ingredient (%)^1)^				
Basal starter/grower^2)^	90.89	92.75	92.79	92.71
Grain sorghum	0.00	7.25	7.21	7.29
Dextrose	9.10	0.00	0.00	0.00
Calculated composition^3)^ (%)				
ME (kcal/kg)	3,198	3,176	3,154	3,154
Crude protein	25.85	27.13	27.06	27.05
Crude fat	9.07	9.45	9.43	9.44
Calcium	0.94	0.96	0.96	0.96
Sodium	0.19	0.19	0.19	0.19
Lysine	1.77	1.82	1.82	1.82
Methionine	0.87	0.91	0.90	0.90
Methionine+cysteine (SAA)	1.26	1.31	1.31	1.31
Total phosphorus	0.91	0.95	0.95	0.96
Available phosphorus	0.66	0.68	0.68	0.68
Analyzed composition^4)^ (%)				
Crude protein	26.97	27.76	28.03	29.22
Crude fat	8.34	8.84	7.83	8.74
Calcium	1.12	1.07	1.11	0.97
Sodium	0.15	0.12	0.15	0.10
Lysine	2.26	2.20	2.14	2.10
Methionine	1.01	0.92	1.01	0.88
Methionine+cysteine (SAA)	1.42	1.35	1.42	1.30
Total phosphorus	0.82	0.86	0.88	0.84

ME, metabolizable energy.

1), 2) Phase-fed basal diet (Table 2) and complete diet formulation based on an industry-standard supplied by a commercial nutritionist.

3) Calculated AME_n_ of each complete test diet determined using equation (1) Treatment AME_n_ [9].

4) Proximate analysis was determined using the AOAC (Association of Official Analytical Chemists) method (Novus International, Inc., Laboratory Services, St. Charles, MO, USA).

**Table 4:** Experiment 1 mean apparent metabolizable energy (AME_n_), and 72-h feed intake by treatment (corn/dextrose, red/bronze, white/tan, and U.S. No. 2 grain sorghum) during the excreta collection period, at 3-wks (19 to 21 d of age) and 6-wks (36 to 38 d of age), in Japanese quail

Items	Age	

	3-wk	6-wk
AME_n_ grain (kcal/kg)		
Dextrose-control	3,106±134.30^bc^	3,106±309.18^ab^
Red/bronze	3,524±122.03^a^	3,373±297.35^a^
White/Tan	3,252±122.03^b^	3,279±297.35^a^
U.S. No. 2	3,039±123.44^c^	2,966±298.64^b^
Main effect	p-value	
Treatment	<0.0001^*^	0.0322^*^
72-h feed intake (kg)		
Dextrose-control	0.496±0.07	0.362±0.03
Red/Bronze	0.496±0.07	0.359±0.02
White/Tan	0.495±0.07	0.341±0.02
U.S. No. 2	0.502±0.07	0.334±0.02
Main effect	p-value	
Treatment	0.9981	0.6211
Source of variation		
Age	0.5147	
Age×treatment	0.8746	

1) LSMeans±standard error; each means represents 10 cages with 5 birds/cage for control, and 18 cages with 5 birds/cage for red bronze, white-tan, and U.S. No. 2.

a–c Means within the same column connected by the same letter are not significantly different.

* Statistical significance: p<0.05.

**Table 5:** Ingredient and nutrient composition of starter/grower Japanese quail treatments (%, as-fed) for Experiment 2 growth performance (1 to 39 d of age)

Items	Dietary treatments			

	Corn	Red/Bronze	White/Tan	U.S. No. 2
Ingredient (%)				
Corn	42.00	0.00	0.00	0.00
Grain sorghum	0.00	43.02	41.62	38.09
Soybean meal, 47.5%	46.00	46.00	46.00	47.00
Fat, vegetable	6.85	6.26	7.67	9.83
Mono-dicalcium phosphate	1.70	1.70	1.69	1.69
Limestone	1.13	1.14	1.14	1.11
Biolys	0.54	0.62	0.63	0.58
Sodium chloride	0.40	0.42	0.41	0.41
DL-methionine	0.50	0.52	0.53	0.54
L-threonine	0.20	0.20	0.20	0.20
Choline chloride, 60%	0.25	0.25	0.25	0.25
Vitamin and trace mineral premix^1)^	0.18	0.18	0.18	0.18
Copper sulfate (5H_2_O)	0.05	0.05	0.05	0.05
Sacox 60^2)^	0.04	0.04	0.04	0.04
Phytase^3)^	0.03	0.03	0.03	0.03
Calculated composition (%)				
ME (kcal/kg)	3,142	3,142	3,142	3,142
Crude protein	26.00	26.00	26.13	26.08
Crude fat	8.85	7.87	9.16	11.34
Calcium	1.26	1.26	1.26	1.26
Sodium	0.18	0.18	0.18	0.18
Lysine	1.66	1.66	1.66	1.66
Methionine	0.83	0.84	0.85	0.85
Methionine+Cysteine (SAA)	1.20	1.20	1.20	1.20
Total phosphorus	0.92	0.92	0.92	0.93
Available phosphorus	0.60	0.60	0.60	0.60
Analyzed composition (%)^4)^				
Crude protein	26.34	26.56	26.70	27.10
Crude fat	8.78	6.86	7.48	7.19
Calcium	1.29	0.94	0.87	1.07
Sodium	0.13	0.13	0.06	0.10
Lysine	1.87	2.03	1.85	1.85
Methionine	0.94	1.02	0.87	0.93
Methionine+Cysteine (SAA)	1.28	1.37	1.23	1.28
Total phosphorus	0.89	0.82	0.74	0.84

ME, metabolizable energy.

1) Commercial vitamin and trace mineral premix for turkeys.

2) Salinomycin sodium, Saccox 60 (Huvepharma, Peachtree City, GA, USA).

3) Ronozyme, HiPhos (DSM, Nutritional Products, LLC, Kingstree, SC, USA).

4) Proximate analysis was determined using the AOAC (Association of Official Analytical Chemists) method (Novus International, Inc., Laboratory Services, St. Charles, MO, USA).

**Table 6:** Effect of treatment (corn, red/bronze, white/tan, and U.S. No. 2) on growth performance responses (BW, FI, and FCR) for Japanese quail 1 to 40 days of age in Experiment 2

Treatment^1)^	BW (kg/bird)	FI (kg/bird)	FCR (kg/kg)	% Mortality
Corn	0.210^b^±0.006	0.543±0.034	2.75±0.178	11.90^c^±3.15
Red/Bronze	0.206^b^±0.005	0.545±0.054	2.76±0.138	22.14^ab^±2.44
White/Tan	0.204^b^±0.005	0.576±0.054	2.93±0.138	22.14^ab^±2.44
U.S. No. 2	0.228^a^±0.005	0.575±0.054	2.81±0.138	25.71^a^±2.44
p-value	0.0031^*^	0.7824	0.8336	0.0181^*^

BW, body weight; FI, feed intake; FCR, feed conversion ratio; SEM, standard error of the mean.

1) LSMeans±SEM; each mean represents 6 pens with 14 birds/pen (kg/bird) for control, and 10 pens with 14 birds/cage (kg/bird) per treatment for red/bronze, white/tan, and U.S. No. 2.

a-c Means within the same column connected by the same letter are not significantly different.

* Statistical significance: p≤0.05.

**Table 7:** Effect of treatment (corn, red/bronze, white/tan, U.S. No. 2) on carcass traits in Japanese quail in Experiment 2

Measurement^1)^	Treatments				p-value

	Corn	Red/Bronze	White/Tan	U.S. No. 2	
LBW (kg/bird)	0.203±0.005^b^	0.208±0.003^b^	0.207±0.003^b^	0.220±0.003^a^	0.0409^*^
HCW (kg/bird)	0.034±0.0008^b^	0.035±0.0006^b^	0.034±0.0006^b^	0.037±0.0006^a^	0.0234^*^
CY (%)	77.89±1.32^a^	77.61±0.877^a^	75.36±0.877^a^	70.33±0.877^b^	<0.0001^*^
BrW (kg/bird)	0.052±0.004	0.052±0.003	0.057±0.003	0.051±0.004	0.4751
BrY (%)	32.78±2.34	32.33±1.82	36.70±1.82	33.16±1.82	0.2800

LBW, live body weight; HCW, hot carcass weight; CY, carcass yield; BrW, breast weight (bone-in); BrY, breast yield; SE, standard error.

1) LSMeans±SE; each means represents 6 replicate pens for 6 birds (kg/bird) per C treatment, and 10 replicate pens of 6 birds (kg/bird) per red/ bronze, white/tan, and U.S. No. 2.

a,b Means within a row connected by the same letter are not significantly different.

* Statistical significance: p<0.05.
